# Smart E-Tongue Based on Polypyrrole Sensor Array as Tool for Rapid Analysis of Coffees from Different Varieties

**DOI:** 10.3390/foods13223586

**Published:** 2024-11-10

**Authors:** Alvaro Arrieta Almario, Oriana Palma Calabokis, Eisa Arrieta Barrera

**Affiliations:** 1 Department of Biology and Chemistry, Universidad de Sucre, Sincelejo 700001, Colombia; eisa.v.arrieta@gmail.com; 2 Faculty of Engineering and Basic Sciences, Fundación Universitaria Los Libertadores, Bogotá 111221, Colombia; opalmac@libertadores.edu.co

**Keywords:** smart e-tongue, polypyrrole, sensor array, coffee

## Abstract

Due to the lucrative coffee market, this product is often subject to adulteration, as inferior or non-coffee materials or varieties are mixed in, negatively affecting its quality. Traditional sensory evaluations by expert tasters and chemical analysis methods, although effective, are time-consuming, costly, and require skilled personnel. The aim of this work was to evaluate the capacity of a smart electronic tongue (e-tongue) based on a polypyrrole sensor array as a tool for the rapid analysis of coffees elaborated from beans of different varieties. The smart e-tongue device was developed with a polypyrrole-based voltammetric sensor array and portable multi-potentiostat operated via smartphone. The sensor array comprised seven electrodes, each doped with distinct counterions to enhance cross-selectivity. The smart e-tongue was tested on five Arabica coffee varieties (Typica, Bourbon, Maragogype, Tabi, and Caturra). The resulting voltammetric signals were analyzed using principal component analysis assisted by neural networks (PCNN) and cluster analysis (CA), enabling clear discrimination among the coffee samples. The results demonstrate that the polypyrrole sensors can generate distinct electrochemical patterns, serving as “fingerprints” for each coffee variety. This study highlights the potential of polypyrrole-based smart e-tongues as a rapid, cost-effective, and portable alternative for coffee quality assessment and adulteration detection, with broader applications in the food and beverage industry.

## 1. Introduction

Coffee is one of the most popular and consumed beverages worldwide, with an estimated 400 billion cups consumed annually [1,2,3]. Because coffee is a lucrative trade, adulteration has become a common practice. Ground roasted coffee is adulterated by adding other components such as by-products of coffee processing (husks and sticks), as well as wheat, corn, barley, soy, rye bran, and others. In addition, inferior quality beans are added, either by using defective beans or other coffee varieties or species [4,5,6].

Therefore, preventing and detecting fraudulent practices is of great importance for the sector, since the quality of coffee is a crucial factor that influences its acceptance and price in the market. Quality assurance in the coffee industry involves techniques that allow knowing its physical, chemical, and organoleptic characteristics, which allows guaranteeing excellent products and preventing fraud [4,7,8,9]. Organoleptic characteristics include color, aroma, and flavor, which are traditionally evaluated by expert tasters through sensory tastings. However, these methods depend largely on the experience and physical condition of the taster, which can lead to variations in the results. In addition, sensory tastings are time-consuming procedures and are not always practical for the analysis of large volumes of samples. On the other hand, chemical characterization methods generally require sophisticated instrumentation such as UV-Vis spectrometers, chromatographs, and FTIR spectrometers, among others [10,11,12,13], in addition to qualified personnel for their operation, which makes them expensive in time and money.

**Figure 1:**
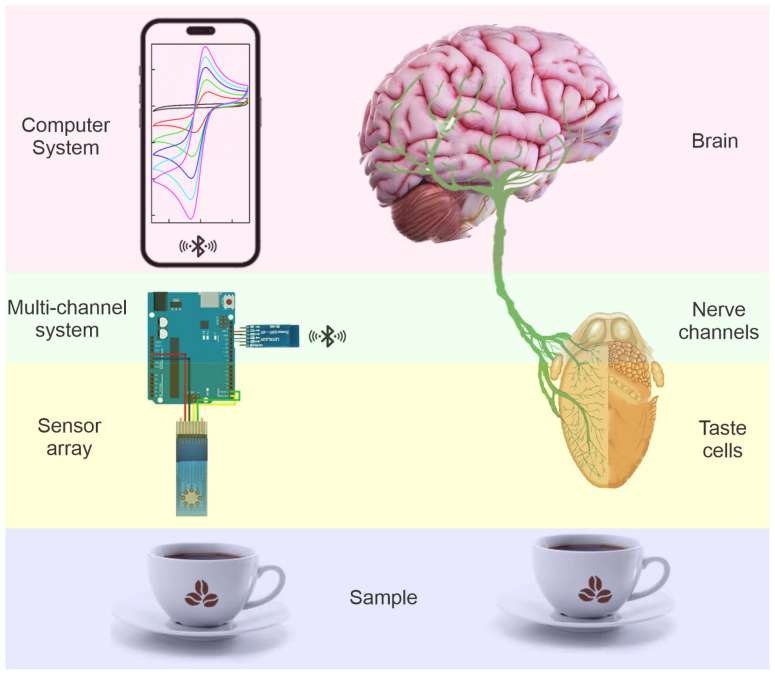
Scheme of functional analogy between the human taste system and an e-tongue and its parts (sample; sensory array; multi-channel system; and pattern recognition system).

In the search for fast, affordable, portable, and reproducible analytical methods, electronic tongues (e-tongues) have emerged as a promising solution. According to IUPAC, an electronic tongue is a chemometric device composed of a sensor array with cross-selectivity, coupled to a multi-channel signal-recording system and pattern recognition software [14]. These devices emulate human taste perception by using non-selective chemical sensor arrays capable of generating response patterns. Through multi-variate statistical methods, they can differentiate, discriminate, and classify complex liquid samples, such as beverages. The term “electronic tongue” is used because of the structural and functional analogy between the human taste system and these electronic systems. Figure 1 presents a scheme showing the functional analogy between the human taste system and an e-tongue. It can be seen that just as in the human taste system, where sensory perception is done through an array of non-selective cells, in the e-tongue, the samples are analyzed using a non-selective sensor array. Once the signals are generated, the human system transmits them to the brain through neuronal channels for interpretation, and it does so through the thalamus. Similarly, in e-tongues, a multi-channel measurement system (for example, a multi-potentiostat) transmits the information to a computer equipped with statistical software for pattern recognition and identification.

Since the first prototype developed by Toko et al. was reported [15], work has been carried out on devices based on different analytical principles. Thus, the development of sensors for e-tongues has advanced considerably in the last decades, with a variety of materials and technologies used. For example, sensor arrays based on fluorescent sensors with soluble conjugated polymeric nanoparticles (SCPN) embedded in water-based polyurethane for tea discrimination have been developed [16], as well as multi-metal sensor arrays (platinum-Pt, gold-Au, palladium-Pd, wolfram-W, titanium-Ti, silver-Ag, iridium-Ir, and rhodium-Rh) for the analysis of drinking water, vinegar, tea, among others [17,18,19]. Enzyme-based colorimetric sensor arrays fabricated with terephthalic acid-modified graphene (TPA-GQD) with metal ions (${Fe}^{2+}$, ${Cu}^{2+}$, ${Zn}^{2+}$) have also been used for the identification of thiols [20]. In addition, conjugated conducting polymers have been used in the fabrication of sensor arrays for the analysis of orange juice, beer, wine, coffee, milk, etc. [21,22,23,24,25], and lipid membrane sensor arrays for the analysis of Fo Tiao Quiang soup, beef, etc. [26,27].

Conducting polymers have shown significant potential in sensor fabrication due to their electrochemical, mechanical, and chemical properties [28]. Polypyrrole (PPy) stands out for its high chemical and thermal stability, ease of synthesis, doping capacity, and electroactivity, making it widely studied in sensor development [29,30,31,32]. The ability to functionalize PPy with various chemical groups and dope it with counterions improves its selectivity and sensitivity toward specific analytes [33,34,35,36]. In this study, electrochemically synthesized PPy was doped with different counterions to fabricate a sensor array capable of distinguishing coffee samples from various bean varieties. Electrochemical synthesis allows precise control of PPy film deposition on electrodes, optimizing sensor performance. Doped PPy sensors demonstrate high chemical stability, easy miniaturization, and enhanced sensitivity to compounds present in complex liquid matrices such as coffee. The unique electroactivity of PPy, combined with its doping versatility, improves the selectivity and performance of the sensor array, which may enable effective differentiation of coffee varieties. This innovative approach can significantly improve traditional detection methods.

This research focuses on the development of an e-tongue based on a PPy sensor array for the rapid analysis of coffees of different varieties. The underlying hypothesis is that PPy sensors, due to their ability to respond to different chemical compounds present in coffee of different varieties, can generate distinctive patterns that allow differentiation between bean varieties. This approach would not only provide an objective and reproducible tool for coffee quality assessment but could also be applied in other fields of the food and beverage industry.

The analysis of the coffee samples was carried out using a voltammetric smart e-tongue based on an array of seven PPy sensors coupled to a portable chemometric system based on PSoC microchip technology controlled by a smartphone. The discrimination capacity of the system was evaluated using samples of the Typica, Bourbon, Maragogype, Tapi, and Caturra varieties. The data matrix was analyzed using principal component analysis assisted by neural networks (PCNN) and cluster analysis (CA), which allowed the visualization and classification of the coffee samples based on the responses of the sensors.

## 2. Materials and Methods

### 2.1. Materials and Reagents

Reagents were purchased from Sigma-Aldrich (St. Louis, MO, USA) and Merck (Darmstadt, Germany). The reagents used in this study included lithium perchlorate (99.99%, Merck), sodium dodecylbenzene sulfonate (99.0%, Aldrich), sodium sulfate (99.0%, Aldrich), ammonium persulfate (98.0%, Merck), potassium ferrocyanide, p-toluenesulfonic acid, anthraquinone-**2**,**6**-disulfonic acid disodium salt (99.9%, Aldrich), and pyrrole (98.0%, Aldrich). Solutions were prepared with Milli-Q ultrapure water (18 MΩ cm^−1^). The coffee samples analyzed consisted of 35 samples: 5 varieties of Arabica coffee (Typica, Bourbon, Maragogype, Tabi, and Caturra) with 7 replicates for each variety. The coffee samples were prepared following the guidelines of NTC 3566 (Colombian Technical Standard for sample preparation for sensory analysis [37]). A total of 7 g of coffee sample was weighed and added to 100 mL of water. The water was heated to boiling point, and the infusion was allowed to steep for 5 min. After, any remaining solids were removed from the surface. Samples were prepared in 7 independent batches, each batch containing one sample from each of the five categories (i.e., bean varieties) of coffee. Samples within each batch were prepared simultaneously, but batches were prepared independently of each other to ensure that there were no correlations between sample preparation and measurements.

### 2.2. Smart E-Tongue

**Figure 2:**
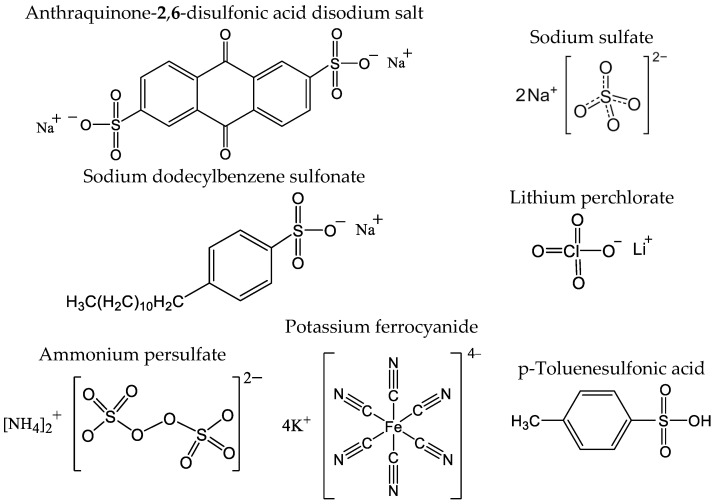
Chemical structure of doping ions used in the preparation of the PPy sensor array.

The smart e-tongue developed in our laboratory was composed of an array of PPy-based voltammetric sensors and a portable multi-potentiostat operated via smartphone. This sensor array included seven electrodes, each composed of PPy doped with distinct counterions: PPy/AQDS (anthraquinone-**2**,**6**-disulfonic acid disodium salt), PPy/SO_4_ (sodium sulfate), PPy/DBS (sodium dodecylbenzene sulfonate), PPy/PC (lithium perchlorate), PPy/SF (ammonium persulfate), PPy/FCN (potassium ferrocyanide), and PPy/TSA (p-toluenesulfonic acid). These compounds used as dopant counterions were selected for providing stability and sensitivity to the sensors because their structures allow stable entrapment in the PPy matrix either by their size or by electrostatic interactions due to their polyionic character or strong polarity. Details of the selection and optimization have been previously published [32]. Figure 2 shows the chemical structures of the dopant counterions used in each of the sensors in the array.

**Figure 3:**
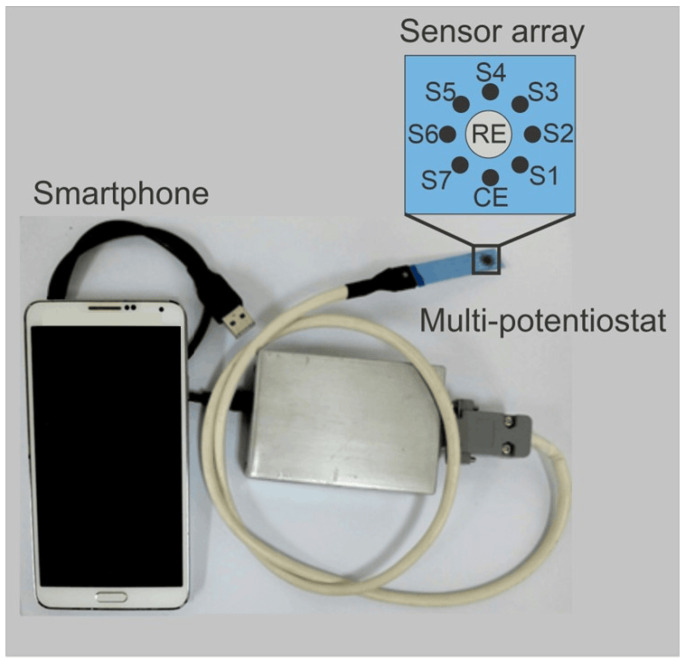
Image of the smart e-tongue device and its parts (sensor array, multi-potentiostat, and data recording and processing system).

The sensors were fabricated using chronoamperometric electropolymerization of pyrrole with an EG&G 2273 PARSTAT potentiostat/galvanostat (Oakridge, TN, USA), managed by PowerSuite software V 2.60. The sensor array was arranged in a circular pattern and fabricated on a commercial AC9C card from BVT Technologies (Strážek 206, 592 53 Strazek, Czech Republic); it consists of an Ag/AgCl reference electrode (RE) and eight platinum electrodes, of which one was used as counter electrode (CE) and seven for the preparation of the seven PPy sensors (S1 to S7) by electropolymerization of pyrrole with different counterions. For the elaboration of each sensor, a PPy solution with a concentration of 0.1 mol L^−1^ and a polymerization potential of 0.8 V was used in all the cases. The specific parameters used for the sensor fabrication are resumed in Table 1. Detailed information on the optimization process for this procedure has been documented in prior publications [36]. The portable multi-potentiostat was created on a FREESOC card using a PSoC 5LP microchip of Mouser Electronics (Mansfield, TX, USA), programmed with PSoC creator software V 4.4. This device recorded the voltammetric signals from the seven sensors simultaneously across seven channels. Additionally, a Bluetooth card enabled data transmission to a smartphone equipped with an Android app for device control and data recording. Figure 3 shows an image of the smart e-tongue device where can be observed the PPy sensor array, the multi-potentiostat, and the smartphone used. The detailed methodologies on electrochemical polymerization and electronic device development have been documented in previous works [32,38,39,40].

### 2.3. Measurement Protocol

Once the samples were prepared, they were allowed to cool down to room temperature to carry out the measurements with the smart e-tongue. Because in the first voltammetric sweeps of the newly prepared sensors, processes of progressive rearrangement of charges in the polymer matrix occur [41,42], it was necessary to carry out some initial stabilization sweeps. In the successive sweeps, the voltammetric signals were stabilized. For this reason, five initial stabilization sweeps were programmed and then the recording of the voltammetric signals already stabilized in the sixth sweep was performed. Coffee samples were evaluated at room temperature using 10 mL per sample. Voltammetric signals were carried out with a scan rate of 100 mV s^−1^, with potential range of −1.0 V to 0.5 V, starting from an initial potential of 0.0 V. The potentials were referenced with the Ag/AgCl electrode (RE) integrated in the sensor array. Measurements were performed randomly in each of the batches to avoid possible correlations between measurement conditions and the resulting sample categories. For the recording of the voltammograms, the sensor array was introduced into 10 mL of sample. Each recording lasted 55 s per sample. The Android application processed the data by recording the currents (I) from the voltammograms and organizing them into a data matrix, where each column represents the current values of the signals.

### 2.4. Statistical Analysis

In the measurements performed, each sensor produced a voltammogram containing 100 current values including the anodic and cathodic sweeps, resulting in a total of 700 columns (7 sensors × 100 data points each), and taking into account the five coffee varieties studied with seven replicates each (samples of 5 varieties × 7 replicates of each sample) resulted in a total of 35 rows. Therefore, the matrix consisted of 24,500 data with 700 columns (variables) × 35 rows (samples). The obtained data matrix was analyzed using principal component analysis assisted by neural networks (PCNN) and cluster analysis (CA). Statistical analysis was performed using Minitab V.19 and statistica V.13.3 software. These techniques have been widely used to carry out the dimensionality reduction of large amounts of data, discrimination, and pattern recognition due to their easy interpretation and low computational cost [43,44]. PCNN is effective at capturing temporal and spatial correlations in complex data, allowing subtle differences to be distinguished. CA complements this process by clustering samples based on similarities, providing a clear visualization of the clusters. PCNN used a network with three layers: input with voltammetric data, output to classify coffee varieties, and 10 neurons in the hidden layer with sigmoid function and Krzanowski cross-validation. In the cluster analysis, complete linkage and Euclidean distance were used. For the visualization of the clustering process, a dendrogram was used, to facilitate the identification of homogeneous groups of coffee samples of different varieties.

## 3. Results and Discussion

### 3.1. Redox Response Mechanism of PPy Sensors

**Figure 4:**
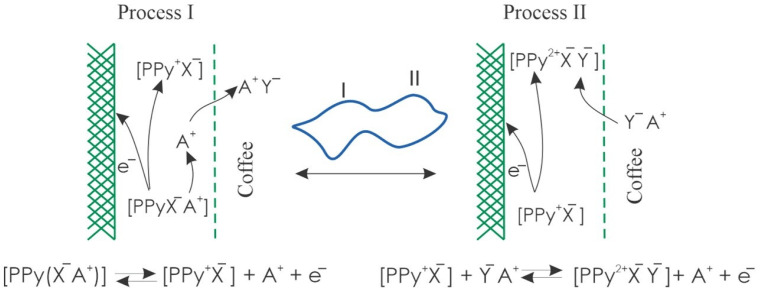
Schematic of oxidation/reduction processes in PPy sensors where cation exchange (process I) and anion exchange (process II) are presented.

The redox processes observed in the sensors were induced by the interaction between the PPy film and the chemical constituents of the coffee samples. Figure 4 illustrates a schematic representation of the charge exchange mechanisms occurring throughout the voltammetric scanning process. Redox processes involved the exchange of charged chemical species with the analyzed medium (i.e., the sample) depending on the oxidation state of the PPy. During the first redox process (process I), an exchange of positively charged chemical species (cations) present in the medium occurred, due to the immobilization of anions in the polymer to maintain electroneutrality. Thus, the cations left when the PPy was oxidized in the anodic sweep and entered when the reduction occurred in the cathodic sweep.

While in the second redox process (process II), the polymeric matrix released negatively charged chemical species (anions) present in the medium to balance the charges. In this way, in the anodic sweep, when the PPy was oxidized for the second time, the anions of the medium entered the polymeric matrix and left in the cationic sweep when the PPy was reduced. This allowed the signals from the sensor array to contain information about each of the coffee samples analyzed. In addition, the voltammetric signals could be influenced by interactions with non-ionic molecules, also present in the coffee samples, such as strongly polar molecules, so the signals were specific to each sample. In this way, the different sensors presented very different behaviors. In this way, the variability in the sensor signals is the product of interactions that can be synergistic or antagonistic between different chemical components. This implies that discrimination between coffee categories is not necessarily due to differences in specific components, but to a complex and composite effect that allows the formation of particular “electrochemical fingerprints”.

### 3.2. Evaluation of the Stability and Repeatability of Voltammetric Responses of Sensors

**Figure 5:**
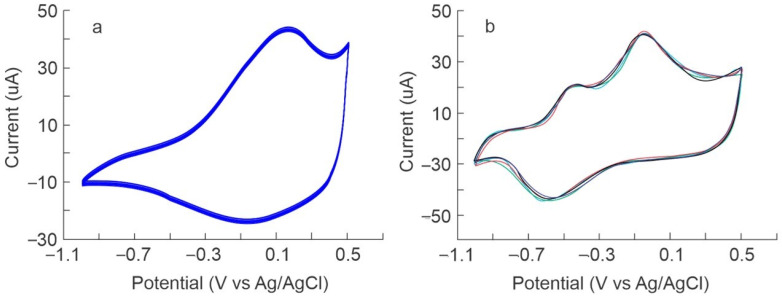
Reproducibility of (**a**) the voltammetric signals of 50 cycles of the S7 sensor (PPy/TSA) against a coffee sample from Maragogype variety beans and (**b**) Voltammetric signals recorded by different S5 sensors (PPy/DBS) in replicates of Typica variety bean coffee prepared in different batches. The lines represent all the replicates of the voltammetric signals of each sensor.

To ensure the stability of the sensors, 50 voltammetric cycles were measured. Figure 5a shows the behavior of the S7 (PPy/TSA) sensor against a coffee sample prepared with Maragogype beans. It can be observed that the signals were stable. The CV coefficient of variation calculated for the sensors was 0.62% for S1 (PPy/PC), 0.89% for S2 (PPy/FCN), 0.97% for S3 (PPy/SF), 0.73% for S4 (PPy/SO4), 0.94% for S5 (PPy/DBS), 0.87% for (PPy/AQDS), and 0.78% for S7 (PPy/TSA).

Furthermore, the reproducibility of the sensor response was evaluated in replicates of coffee samples. For this purpose, five replicates of coffee samples of the same bean variety prepared in different batches and five replicates of the sensor array were used. This ensured the reproducibility of the preparation of the replicates of the samples in the batches and of the preparation of the sensors. Figure 5b shows, as an example, the responses of the S5 sensor (PPy/DBS) against replicates of samples prepared with Typica coffee from different preparation batches. It can be observed that there are no significant differences in the signals and that the response was homogeneous, which ensured that the variabilities recorded by the sensor array were not due to variability in the preparation of the samples or the sensor array.

### 3.3. Assessment of the Cross-Selectivity of the Sensor Array

**Figure 6:**
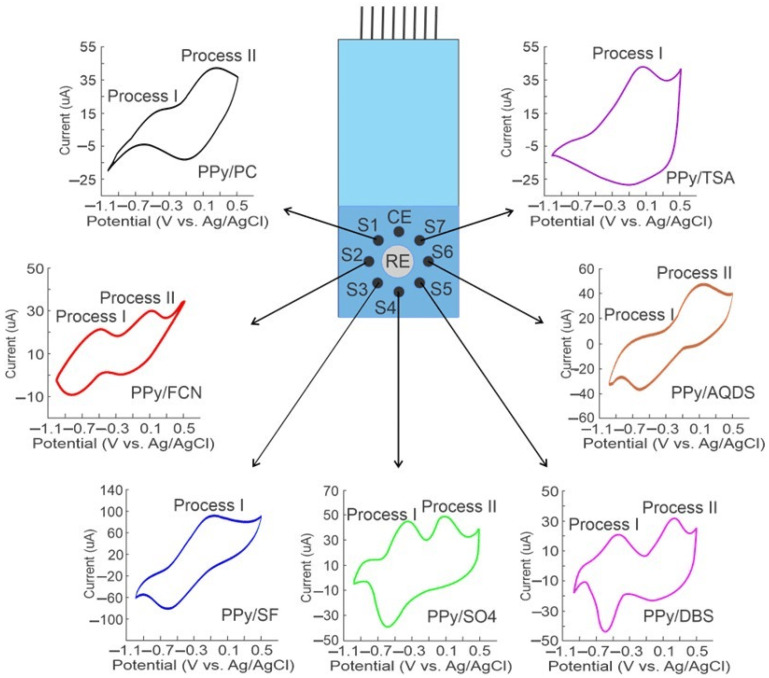
Voltammetric signals recorded by the sensor array against a coffee sample prepared with Caturra variety beans as instance of sensor redox response variability that contributes to cross-selectivity.

Once the reproducibility of the measurements was assessed and verified, the cross-selectivity of the PPy sensor array was evaluated. Figure 6 shows as an example the voltammetric signals of the sensor array recorded in a Caturra coffee sample. The signals exhibited redox processes that were related to the oxidation/reduction of PPy in response to an interaction with the sample.

As shown in Figure 6, the voltammetric signal of S1 (PPy/PC) displays a pair of anodic peaks at −0.48 V (process I) and 0.29 V (process II); in the cathodic sweep, only a single reduction peak at −0.09 V is clearly observed. The signal recorded with S2 (PPy/FCN) consisted of two redox transformations. The redox process designated as I presented an anodic peak at −0.51 V, paired with a cathodic peak that was seen at −0.83 V, and process II with an anodic peak at 0.98 V and its cathodic pair at −0.21 V. In this case, the second process was due to the transformation of ferrocyanide ${\left[Fe(CN\right)}_{6}^{3-}/{Fe(CN)}_{6}^{4-}$] within the polymeric matrix. The response of S3 (PPy/SF) showed only a redox process, with a pronounced oxidation peak at −0.09 V and a reduction peak in the cathodic scan at −0.59 V. The voltammetric responses of S4 and S5 (PPy/SO4 and PPy/DBS, respectively) showed in both cases a pair of redox processes, composed of two anodic peaks at −0.34 V and 0.11 V for PPy/SO4 and −0.46 V, and 0.26 V for PPy/DBS. In the cathodic scan, it was observed that PPy/SO4 presented only the reduction peak of process I at −0.60 V. On the other hand, the cathodic scan of PPy/DBS showed in processes I and II well-defined reduction peaks at −0.57 V and −0.02 V.

**Figure 7:**
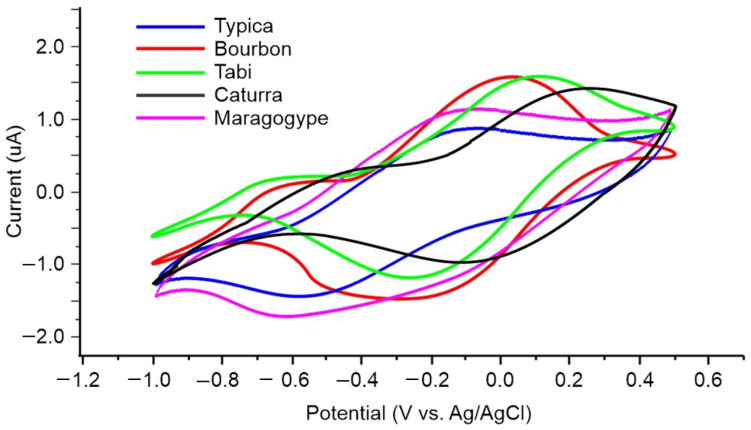
Voltammetric signals of the lithium perchlorate/S1 doped sensor (PPy/PC) versus coffee samples prepared with beans of different varieties as instance of response variability and cross-selectivity.

The voltammetry recorded with S6 (PPy/AQDS) included the redox processes composed of two anodic oxidation peaks at −0.55 V and 0.08 V and two peaks in the cathodic scan due to the reduction at −0.63 V and 0.06 V. S7 showed an oxidation process in the anodic scan at 0.07 V and a broad reduction peak in the anodic scan at −0.08 V. Thus, the shape and position of the peaks in the voltammetric signals were markedly different for each of the sensors of PPy. Table 2 summarizes the peak potentials and currents of the redox processes (oxidation/reduction) of the signals presented in Figure 6. Therefore, the variety of responses obtained indicated that each of the sensors provided information on each coffee sample, so the information obtained from the sensor array could allow the recognition of the different coffee samples analyzed. In addition, each sensor generated a different response to each of the samples. Figure 7 shows as an example the response of S1 (PPy/PC) to the different coffee samples prepared with beans of different varieties. As can be seen, the voltammetric signal is markedly different for each coffee sample, so the sensor array presents a high degree of cross-selectivity and the recorded signals can be considered a “fingerprint” of the samples analyzed. According to the IUPAC report, cross-sensitivity refers to the ability of a non-selective sensor array to react to various analytes present in a complex sample [14]. Thus, the redox peaks recorded in the Tabi coffee sample were at −0.11 V for the oxidation process in the anodic scan and −0.52 V for the reduction in the cathodic scan.

Caturra and Maragogype coffee samples presented in the anodic sweep the peaks at 0.23 and −0.10, respectively, with their corresponding reduction pairs in the cathodic wave at −0.09 V and −0.62 V. Meanwhile, the Typica coffee sample presented the oxidation and reduction peaks at −0.08 V and −0.52 V and the Bourbon coffee sample presented them at 0.02 V and −0.24 V. The voltammograms indicated that the electrochemical properties of coffee had a noticeable impact on the location and intensity of the peaks associated with PPy electroactivity. In this way, each of the samples was characterized by presenting a particular signal that contains information about each of the coffee samples analyzed. Therefore, it was possible to extract the information contained in the signals supplied by the sensor array of the smart e-tongue.

### 3.4. Evaluation of Discrimination Capacity of Smart E-Tongue Against Coffee Samples Made with Different Bean Varieties

The voltammetric curve data provided by the PPy sensor array signals obtained from the measurement in the coffee samples were collected in a data matrix. The data matrix was used to carry out the principal component analysis assisted by neural networks (PCNN). This method allows to estimate, through the correlation circle (loadings graph), the effectiveness and quantity of the information provided by each sensor. In addition, it allows, through the scores graph, to evaluate the capacity of the smart e-tongue to carry out the discrimination of coffee samples prepared with beans of different varieties from the information captured by the sensor array in the voltammetric signals.

**Figure 8:**
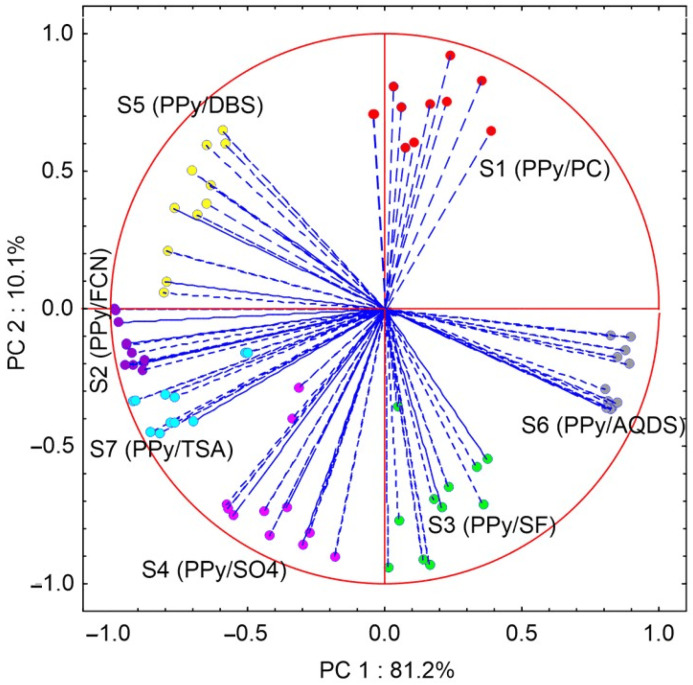
Loading graph resulting from PCNN analysis on the data matrix recorded with the smart e-tongue on the analyzed coffee samples: red-S1 (PPyPC); purple-S2 (PPyFCN); green-S3 (PPy/SF); fuchsia-S4 (PPy/SO4); yellow-S5 (PPy/DBS); grey-S6 (PPy/AQDS); and blue-S7 (PPy/TSA).

Figure 8 shows the correlation circle resulting from the application of PCNN on the data matrix obtained from the records carried out with the smart e-tongue on the coffee samples. The loadings of the first two principal components (PC 1 and PC 2) collect a variance (information) of 91.3%; the first principal component summarizes 81.2% and the second principal component 10.1%. It can be seen that each of the sensors provided effective and sufficient information to carry out the discrimination of coffee samples of different varieties. S1 (PPy/PC) is located in the positive quadrants of both axes (+/+), the S5 sensor (PPy/DBS) is situated in the positive quadrant of the abscissa axis (PC 1) and negative of the ordinate axis PC 2 (+/−). On the other hand, the S2 sensor (PPy/FCN) is laid in the negative quadrant of the abscissa and divided between the positive and negative parts of the ordinate PC2. The S4 (PPy/SO4) and S7 (PPy/STA) sensors are located in the negative quadrant of both axes (−/−). The S3 (PPy/SF) and S6 (PPy/AQDS) sensors are located in the negative quadrant of the abscissa and positive quadrant of the ordinate (−/+). The amount of information is evaluated by the distance from the center of the plane, being smaller when it approaches zero and larger when it approaches 1.0, which is the radius of the circle. In the correlation circle obtained, it can be seen that all the sensors are close to 1.0, which indicates that all of them provide significant information for the discrimination of the samples.

On the other hand, the distribution in the different quadrants of the plane in the correlation circle confirms the effectiveness and variety of the information (cross-selectivity) captured by the voltammetric signals. Moreover, it was evident that the sensors did not produce redundant information, as indicated by the limited overlap of points within the circle. This suggests that each sensor contributes valuable insights into the analyzed samples.

**Figure 9:**
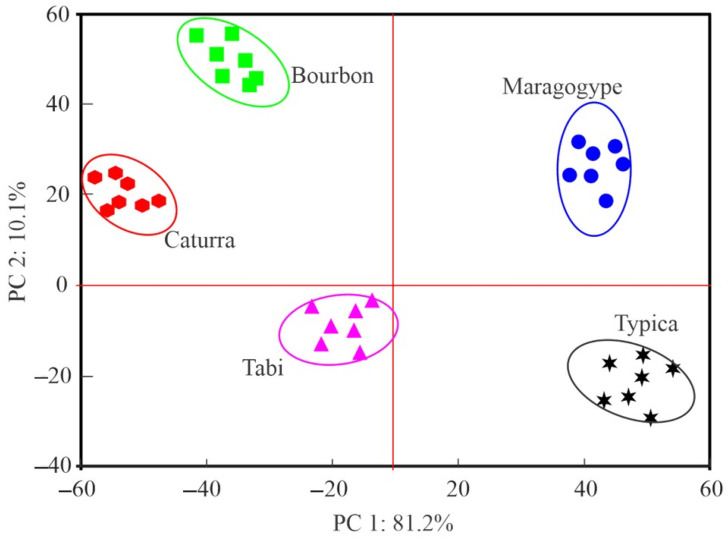
Score graph resulting from the PCNN analysis on the data matrix recorded with the smart e-tongue on the analyzed coffee samples.

The score graph obtained from the application of PCNN to evaluate the discrimination capacity of the smart e-tongue on coffee samples of different varieties is presented in Figure 9. It can be seen that the samples were noticeably separated into clusters, which corresponded to a 90% confidence interval. The coffee samples prepared with beans of the Maragogype variety were located in the positive quadrant of both components (PC 1 and PC 2). The coffee samples prepared with the Caturra and Bourbon varieties were located in the negative quadrant of the first component and the positive quadrant of the second. The samples prepared with beans of the Tabi variety were shown in the negative quadrants of both principal components, and those prepared with beans of the Typica variety were located in the positive quadrant of the first component and the negative quadrant of the second. The separation of the coffee samples elaborated with beans of different varieties into well-differentiated clusters can be observed in the score graph. In this way, each sample group was well-distinguished, demonstrating that the smart e-tongue successfully classified each sample.

**Figure 10:**
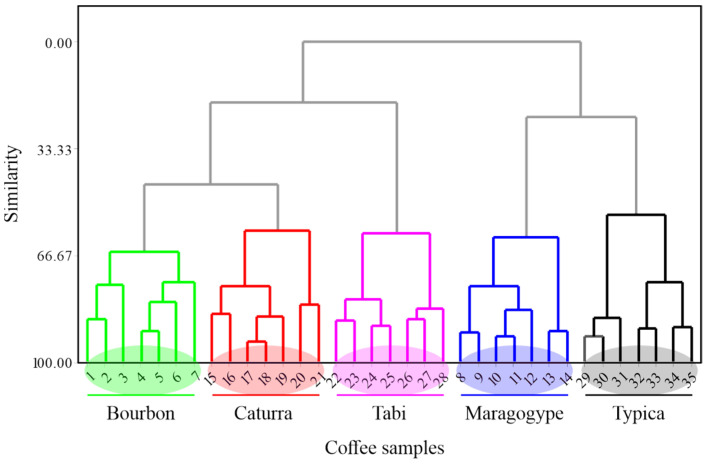
Dendrogram of cluster analysis for coffee samples elaborated with beans of different varieties.

To confirm the accuracy of the results obtained from the principal component analysis, a cluster analysis was carried out. Figure 10 shows the dendrogram resulting from applying cluster analysis to the data matrix from the smart e-tongue measurements.

As can be seen, there was clear discrimination between each of the coffee samples prepared from different bean varieties. In the dendrogram, the samples were grouped into five distinct categories: Bourbon, Caturra, Tabi, Maragogype, and Typica. The Bourbon variety samples exhibited a high degree of similarity, as indicated by their close clustering, suggesting consistent characteristics within this variety. Caturra, although also presenting a tight cluster, showed a slight dispersion compared to Bourbon. The Tabi samples clustered intermediately between those of Caturra and Maragogype, indicating that they could share certain characteristics. Maragogype, like Bourbon, showed a high internal similarity, while Typica, although maintaining an acceptable cohesion, showed a slight dispersion that could indicate variability in the characteristics measured within this variety. The analysis demonstrated the effectiveness of the smart e-tongue to discriminate between different coffee varieties, allowing not only the precise identification of each variety but also the evaluation of the consistency within each group. Furthermore, the observed relationship between sample groups was consistent with the spatial distribution obtained from the PCNN analysis.

## 4. Conclusions

This work successfully demonstrated the effectiveness of a smart e-tongue based on a PPy sensor array for the rapid analysis of coffee samples from different varieties. The developed smart e-tongue, featuring a voltammetric sensor array integrated with a portable multi-potentiostat controlled via a smartphone, was capable of distinguishing between different coffee varieties through distinct voltammetric signals. The specific redox processes observed in each sensor indicated that the PPy sensors exhibited high cross-sensitivity versus coffee samples, resulting in unique electrochemical signatures, or “fingerprints”, for each variety. The analysis of these voltammetric fingerprints, using principal components assisted by neural networks (PCNN) and cluster analysis (CA) methods, confirmed the e-tongue ability to effectively classify and discriminate between the different coffee varieties. This study highlights the potential of PPy-based smart e-tongues as a cost-effective and portable tool for the rapid assessment of coffee quality, offering a viable alternative to traditional sensory evaluation methods and complex chemical analyses. This approach not only ensures objective results but also opens up possibilities for broader applications in the food and beverage industry, where quick and accurate quality control is essential.

## Figures and Tables

**Table 1:** Experimental parameters used for the development of the PPy sensor array by electrochemical synthesis through chronoamperometry.

Sensor	Acronym	Counterion Concentration [mol L^−1^]	Polymerization Time (s)
S1	PPy/PC	0.05	60
S2	PPy/FCN	0.1	45
S3	PPy/SF	0.05	70
S4	PPy/SO4	0.1	50
S5	PPy/DBS	0.1	60
S6	PPy/AQDS	0.1	60
S7	PPy /TSA	0.05	70

**Table 2:** Summary of the oxidation/reduction potentials observed in the voltammetric response of PPy sensors against coffee samples prepared with beans of the Caturra variety presented in Figure 1.

Sensor	Acronym	Redox Process I		Redox Process II	
		Oxidation	Reduction	Oxidation	Reduction
S1	PPy/PC	−0.48	-	0.29	−0.09
S2	PPy/FCN	−0.51	−0.83	0.98	−0.21
S3	PPy/SF	-	-	−0.09	−0.59
S4	PPy/SO4	−0.33	−0.60	0.11	-
S5	PPy/DBS	−0.46	−0.57	0.26	−0.02
S6	PPy/AQDS	−0.55	−0.63	0.08	0.06
S7	PPy /TSA	-	-	0.07	−0.08

## Data Availability

The original contributions presented in the study are included in the article, further inquiries can be directed to the corresponding author.
